# Artificial intelligence-driven 3D surface-topography app for screening and monitoring adolescent scoliosis: early results from a single institution

**DOI:** 10.1007/s43390-026-01282-5

**Published:** 2026-01-30

**Authors:** Stefan Parent, Marjolaine Roy-Beaudry, Justin Dufresne, Rachelle Imbeault, Soraya Barchi, Marie Beauséjour

**Affiliations:** 1 Centre de Recherche Azrieli du CHU Sainte-Justine, Montréal, QC Canada; 2 Division of Orthopaedics, Department of Surgery, CHU Sainte-Justine, Montréal, QC Canada; 3 Department of Surgery, University of Montréal, Montréal, QC Canada; 4 Department of Community Health Sciences, Université de Sherbrooke, Sherbrooke, Canada

**Keywords:** Adolescent idiopathic scoliosis, Cobb angle, 3D surface topography, Artificial intelligence, Radiation-free monitoring, Digital health

## Abstract

**Purpose:**

Radiation-free tools, such as scoliometers, ultrasound, and Moiré topography, have been explored for monitoring Adolescent Idiopathic Scoliosis (AIS), but none have replaced the need for serial spinal radiographs. This study aimed to evaluate the accuracy and criterion validity of a new AI-powered digital health application using 3D surface topography to predict Cobb angles, with the goal of reducing radiation exposure and enabling home-based curve monitoring.

**Methods:**

In a single-center observational study, 125 patients with confirmed or suspected scoliosis underwent smartphone-based 3D surface-topography scans in standing and forward-bending positions. Poor-quality scans (*n* = 20) were excluded. Radiographic Cobb angles were used as the gold standard. After random allocation, 79 scans formed the training set and 26 formed the validation set; external data (142 controls, 188 scoliosis patients) were added to strengthen training, and 25 controls were added to the test set. Accuracy was expressed as mean absolute error (MAE) and correlation with radiographs. Criterion validity was assessed by sensitivity, specificity, and AUC at clinically meaningful thresholds (10°, 25°, and 40°).

**Results:**

Across 51 test scans (AIS + controls), the algorithm showed a strong correlation with radiographs (*r* = 0.922, 95% CI 0.866–0.955) and an MAE of 5.9° (95% CI 4.5–7.3). In AIS-only curves of 10–50°, the MAE was 6.4° (95% CI 4.4–8.3). At 10°, sensitivity was 0.962, specificity was 0.960, and AUC was 0.978. At 25°, sensitivity was 0.706, specificity was 0.853, and AUC was 0.917. At 40°, sensitivity was 0.667, specificity was 1.000, and AUC was 1.000.

**Conclusion:**

This AI-driven 3D surface topography demonstrated high validity for non-radiographic Cobb angle prediction, particularly in mild-to-moderate AIS. It supports safer, more frequent, home-based monitoring, though refinements are needed for severe curves and patients with a higher BMI.

**Supplementary Information:**

The online version contains supplementary material available at 10.1007/s43390-026-01282-5.

## Introduction

Adolescent Idiopathic Scoliosis (AIS) is the most prevalent spinal deformity affecting 1–3% of children aged 10–16 years [1, 2]. Current screening methods, including radiation-free techniques such as scoliometers, spinal ultrasound, and Moiré topography, have provided useful clinical information but remain limited as standalone tools. For example, scoliometers only capture a single trunk measurement with modest correlation to radiographic Cobb angles [8–10], and Moiré requires specialized equipment not available in every center and cannot be used in bending positions [11], while ultrasound is highly operator-dependent and less accurate in severe deformities (> 40–50°) [12–14]. Consequently, these modalities often necessitate complementary hospital-based assessments and have not replaced the need for serial spinal X-rays [3–7]. Among the various digital solutions that were proposed to enable the detection, measurement, and monitoring of scoliosis curves [15], surface topography is a technique that analyzes local deviations of the body surface, allowing for 3D modeling of the shape of the back [16]. Compared with radiographic measurements, the use of surface-topography systems has shown correlations ranging from 0.68 to 0.9, [17] with higher correlations observed for thoracic measurements compared to lumbar measurements [16]. Nevertheless, surface topography was reported to provide highly reliable measurements, with intraclass correlation coefficients greater than 0.8 [18].

The purpose of such modalities was to decrease the number of serial spinal X-rays and to detect patients with clinically important spinal deformities [19]. The limitations associated with most of these technologies include the search for a linear relationship between the external trunk measurement and the spinal deformity. This results in an increase in inadequate referrals for scoliosis and, at the same time, an increase in late referrals for appropriate treatment of scoliosis [20].

The integration of artificial intelligence (AI) algorithms into a surface-topography system has demonstrated effectiveness in classifying scoliosis, with an accuracy reaching 87.5% [21]. The authors reported that using a deep learning model to predict the Cobb angle was less satisfactory for more severe curvatures. Moreover, overestimation for mild angles and significant variability for larger angles were observed [17].

AI algorithms may be better suited at identifying specific relationships that may not be linear based on their use of non-linear methods. A new digital health application (*Momentum Health, Montreal, Quebec, Canada*) leverages advanced 3D surface topography technology coupled with AI to predict scoliotic Cobb Angles using a smartphone.

The objective of this study is to validate the accuracy and predictivity of this new technology. Our hypothesis is that imaging technology can be used to accurately predict the curve magnitude of spinal deformity.

## Methods

One-hundred and twenty-five scoliosis patients were recruited for this study over a period of 12 months. Patients with a confirmed radiographic diagnosis of Idiopathic Scoliosis (IS) aged 8–18 years were eligible for inclusion in the study; exclusion criteria included the presence of another musculoskeletal deformity, a neuromuscular disorder, and a history of spine or thoracic surgery. Orthotic braces were removed at least 2 h prior to the acquisition of the radiographs and the topographic scans.

Following their routine AIS follow-up, including full spine PA and lateral X-rays, patients and their legal guardians underwent the informed consent (and assent, if applicable) process with the research coordinator. Topographic scans were performed with the phone app by the research coordinator or a trained research assistant.

From the operators’ perspective, a scan consists of a simple, 30–45 s video taken on a regular smartphone. The user performs three full rotations around the patient, gradually lowering the camera from the thorax to the abdomen and finally to the lower limbs with each pass, to acquire the necessary data. The mobile application provides live, on-screen instructions to the user to facilitate the process, such as recommended distance from the patient and rotation speed, as well as adequate room lighting. Patients were scanned in both the standing and Adam’s Forward Bending Test positions [22]. For the bent forward scans, patients were instructed to aim for 90 degrees of hip flexion, although the hip flexion angle is automatically corrected by the software. The same iPhone^™^ 12 Pro was used for the full data collection protocol.

### 3D model generation and scaling

The personalized 3D model was generated by software extraction of images from selected frames of the video captured with a smartphone. Camera alignment was subsequently performed automatically to build the 3D model. For privacy protection, the patient’s head was digitally cropped from the model. This resulting 3D model combined both generated models: the standing position and Adam’s forward-bending position. The model was finally scaled to obtain a standardized input for the AI model.

### Core 3D model input to the model

Back surface data were extracted from bent scans to enhance the accuracy of left–right asymmetry measurement. The bent scans are initially smoothed and cropped, and the back surface is extracted from the bent scan. This provides a topographical representation of the back surface where the differences in depth between the left and right sides are calculated.

### Additional volumetric input into the model

Standing scans are sliced horizontally into 20 evenly distributed sections, and the volumetric shift of each quadrant is recorded. Gaps caused by the arms are averaged using an elliptical function, and the boundary is also averaged across the height of the slice.

### Architecture of the AI Cobb angle prediction with volumetric embeddings

The architecture begins with X-ray images that have been measured three times and averaged to reduce measurement error (with a noted inter-observer variability of 4° between our center’s annotation and internal annotators from the developer). These X-rays are processed through a Convolutional Neural Network (CNN) composed of multiple convolutional layers. The CNN output is combined with volumetric data collected from the 20 spinal sections, including centroid and quadrant volumes. This combined information feeds into a Random Forest Regressor that processes the data through multiple decision trees and ultimately outputs a maximum predicted Cobb angle. This hybrid approach leverages both image processing and volumetric spatial information to improve prediction accuracy for this important spinal curvature measurement.

The final training dataset comprised 409 three-dimensional surface-topography scans: 142 control scans obtained from patients presenting with minor fractures at an outside orthopedic clinic, and 267 adolescent idiopathic scoliosis (AIS) scans—79 acquired at our institution and 188 sourced from collaborating centers. Curve severity was evenly represented among the AIS cases, with 39 curves < 15°, 60 curves 15–25°, 80 curves 25–40°, and 84 curves > 40°.

Once the model was trained on this cohort, it was validated on an independent hold-out set of 51 scans consisting of 26 AIS patients from our center and 25 control patients.

The results were compared to AP and lateral radiographs obtained the same day. The Cobb angle was measured by an experienced spine surgeon and blinded to the app's prediction. We computed the mean absolute error (MAE) as a measure of accuracy, and criterion validity was tested using sensitivity, specificity, and area under the curve (AUC) for selected meaningful Cobb angle thresholds of 10°, 15°, 25°, and 40°. We additionally calculated the root‑mean‑square error (RMSE) with a 95% confidence interval derived from 10 000 non‑parametric bootstrap resamples.

Proportional bias was assessed with a Bland–Altman regression of the signed differences on their means; significance of the slope was evaluated with a two‑tailed t test (*α* = 0.05).

## Results

Of the 125 patients recruited, 20 poor-quality scans were discarded due to movement during the scan or hair and clothing obstructions; 79 scans were randomly assigned to the training set and 26 to the validation set. To balance both sets, 142 control patients and 188 additional scoliosis patients were incorporated into the training cohort, while 25 control patients were added to the test cohort.

**Fig. 1:**
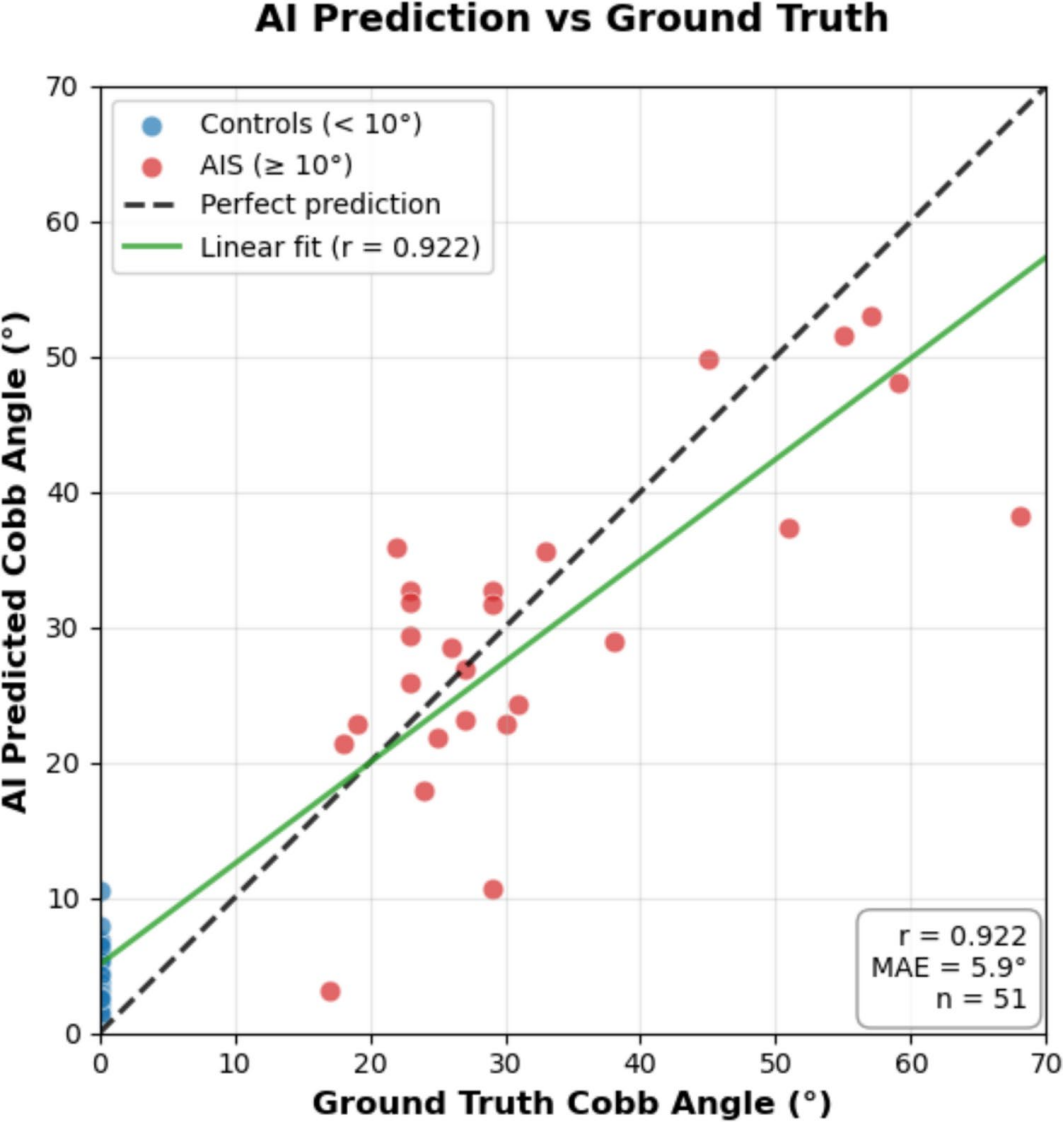
Prediction of Maximum Cobb angle compared to actual measurements

The algorithm predicted Cobb angle (below 50°) with an overall correlation coefficient of 0.922 (95% CI 0.866–0.955**)** and a mean absolute error (MAE) of 5.9° (95% CI 4.5°–7.3°) and an RMSE of 7.75° (95% CI 5.55°–10.12°) when all 51 test scans (AIS + controls) were analyzed (Fig. 1**).** Excluding the 25 controls and evaluating only the AIS scans between 10 and 50**°**, MAE increased slightly to 6.4° (95% CI 4.4°–8.3°).

The screening performance of the app at the 10° threshold showed a sensitivity of 0.962 (95% CI 0.811 – 0.993), a specificity of 0.960 (95% CI 0.805–0.993), and an area under the curve (AUC) of 0.978 (95% CI 0.938 – 1.00). At 25°, the usual threshold for initiating brace interventions, sensitivity was 0.706 (95% CI 0.469 – 0.867)**,** specificity 0.853 (95% CI 0.699 – 0.936**)**, and AUC 0.917 (95% CI 0.821 – 1.000). At 40°—a threshold for which non-surgical treatment has limited effect—sensitivity was 0.667 (95% CI 0.300 – 0.903**)**, specificity 1.00 (0.921 – 1.00**)**, and AUC 1 (1.00 – 1.00).

As can be noted in Fig. 1, the predictions tend to overestimate the measured Cobb angle for smaller curves, whereas they tend to underestimate the Cobb angle for larger curves (over 40°).

**Fig. 2:**
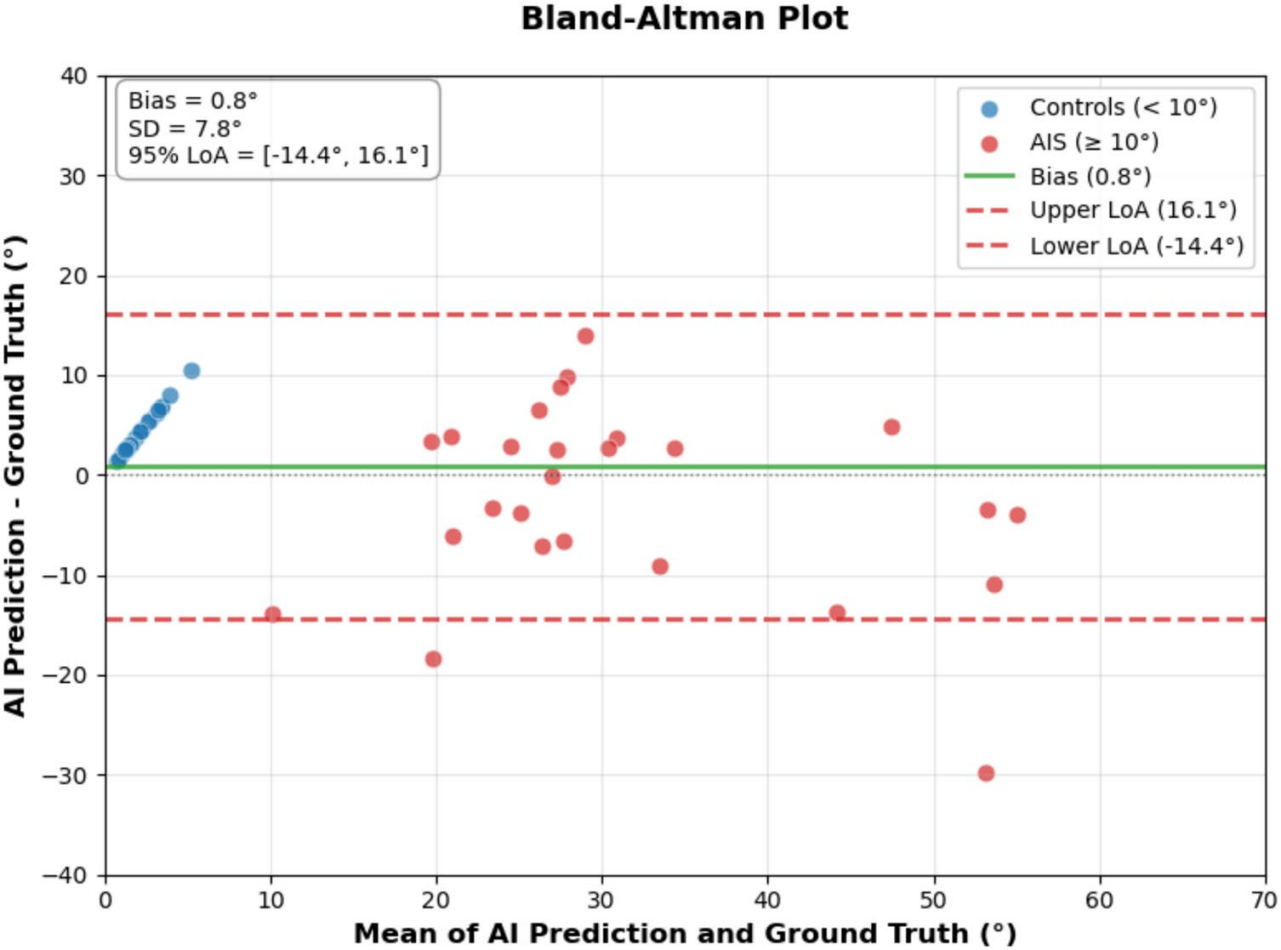
Bland–Altman plot

Bland–Altman analysis demonstrated a mean bias of 0.83° (95% CI –0.67° to 2.32°). A small but statistically significant proportional bias was observed (slope = −0.22° per degree, *p* = 0.00038), indicating slight over‑estimation of small curves and under‑estimation of curves above 40° (Fig. 2).

BMI data were available for (25/26) AIS test patients. Mean BMI was 18.8 ± 2.6 kg/m^2^ (range 15.1–25.9). Analysis revealed no significant correlation between BMI and prediction accuracy (Pearson r = 0.154, *p* = 0.464). Mean absolute error across BMI tertiles was 6.1° ± 3.9° (low BMI: 15.1–17.3), 7.4° ± 6.3° (medium BMI: 17.7–20.0), and 9.6° ± 8.9° (high BMI: 20.1–25.9) with no significant difference between groups (ANOVA *p* = 0.568) (Table 1).

**Table 1:** BMI analysis in test set

Parameter	Value
Patients with BMI data, *n* (%)	25 (49%)
Mean BMI, kg/m^2^	18.8 ± 2.6
BMI range, kg/m^2^	15.1–25.9
Correlation with MAE (*r*, *p*)	0.154, *p* = 0.464
Mean MAE by BMI tertile, degrees	
Low (15.1–17.3)	6.1 ± 3.9
Medium (17.7–20.0)	7.4 ± 6.3
High (20.1–25.9)	9.6 ± 8.9
ANOVA *p* value	0.568

*MAE* mean absolute error

### Model performance

**Fig. 3:**
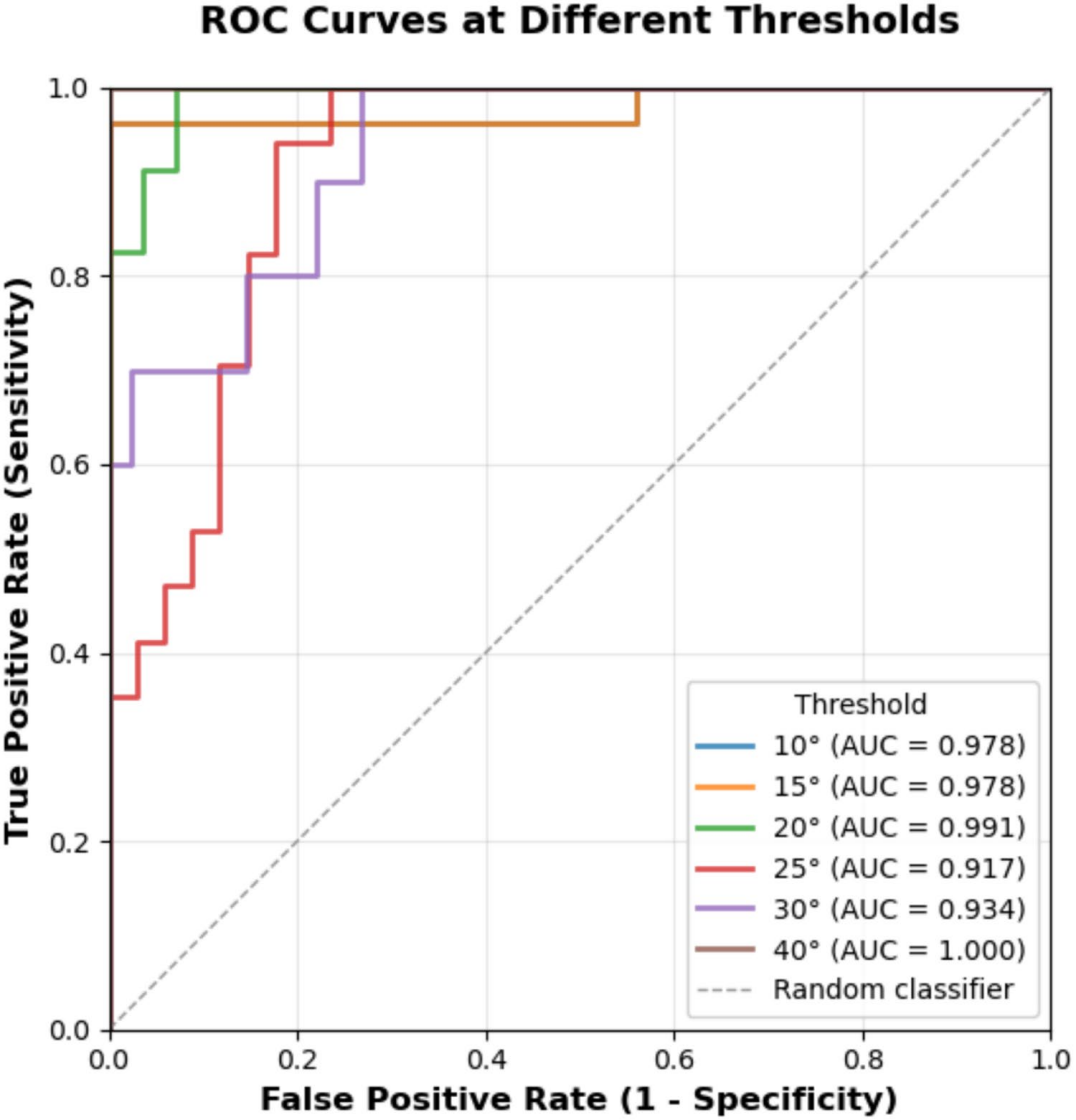
Model performance for different curve severity thresholds

The model's performance was evaluated at four different Cobb angle thresholds: 10°, 15°, 25°, and 40° (Fig. 3). At lower angles (10° and 15°), the model shows strong sensitivity (0.962 and 0.923), good specificity (0.96 and 1.00), and excellent AUC values (0.96 for both). However, as curve severity increases, sensitivity decreases notably, dropping to 0.706 at 25° and 0.667 at 40°, while specificity improves, with a specificity of 0.853 at 25 and 1.00 at 40°. Percent agreement values for the same clinical thresholds are summarized in Table 2.

**Table 2:** Percent agreement between predicted and measured Cobb angles

Threshold (°)	TP	FP	FN	TN	% Agreement
10	25	1	1	24	96.1%
15	24	0	2	25	96.1%
25	12	5	5	29	80.4%
40	4	0	2	45	96.1%

## Discussion

Scoliosis screening has long been recommended to enable early detection and timely referral, with the ultimate goal of improving treatment outcomes. A major limitation of traditional approaches, however, has been the poor correlation between trunk rotation and true spinal deformity, which has led to both unnecessary referrals and delayed diagnoses [3–7]. In this context, our new AI-driven smartphone application offers a radiation-free alternative to quantify external trunk deformities and changes in body shape. This tool has the potential to complement other screening methods and to reduce the frequency of radiographs in the routine care of AIS patients. Given that the prevalence of AIS in the general population is estimated at 2–3%, the pre-test probability remains relatively low. Accordingly, any effective screening tool must prioritize sensitivity over specificity to minimize missed cases. Our model performed well at lower curve severities, achieving high sensitivity at 10° (93%) and 15° (90%), making it particularly suitable for screening. However, sensitivity declined at higher thresholds, such as 25° (78%), limiting its applicability for detecting more advanced deformities. It should be noted that more severe curves should not be self-monitored at home but should be monitored clinically by an experienced clinical team treating spinal deformities.

The clinical management of AIS currently relies on physical examination and radiographic imaging, with follow-up intervals calibrated to balance the risk of curve progression against cumulative radiation exposure. This concern is particularly relevant in AIS patients, who are young and may undergo repeated imaging over several years, raising the long-term risk of radiation-induced malignancies, including breast cancer, due to the radiosensitivity of developing tissues [1, 2, 19, 23–26]. At the same time, rapid curve progression during the pubertal growth spurt can lead to missed opportunities for timely intervention if follow-up intervals are extended. The integration of a radiation-free tool capable of assessing trunk morphology at home could help bridge this gap. By enabling parents or caregivers to monitor progression between visits, such a device could support earlier detection of rapid curve worsening while limiting unnecessary radiographs and clinic visits. In this way, stable patients might safely benefit from less frequent monitoring, whereas those with signs of accelerated progression could be promptly recalled for imaging and treatment, ultimately supporting more adaptive and personalized care strategies. A study is currently underway to estimate the reliability and validity of the tool in the hands of lay observers (responsible adults/caregivers of young IS patients). In addition, the number of X-rays that could have been avoided using the app will be investigated in a clinical trial.

The screening performance of the app showed sensitivity ranging from 0.62 to 0.93, specificity ranging from 0.87 to 1.0, and an area under the curve (AUC) between 0.93 and 0.96; with an overall MAE of 5.9°. The drop in sensitivity at higher angles may be attributed to the relatively limited number of severe curves available during model training. Future work may plan to address this imbalance by exploring stratified or severity-specific models, allowing separate algorithms to be applied to mild/moderate versus high-curvature cases. Other methods combining AI and surface topography have led to the following performance results. Minotti et al [17]. reported a mean absolute error in predicting the Cobb angle of 6.1° ± 5.0°. Moderate correlation (*r* = 0.68) and a root-mean-square error of 8° between the predicted and true values were reported. The overall accuracy in classifying scoliosis severity was 59% and was considered inadequate. Colombo et al [21]. reported an accuracy and a balanced accuracy of the best supervised model close to 85%, giving a low percentage of unclassified patients. The overall RMSE of 7.75° compares favorably with other surface‑topography algorithms (e.g., Minotti et al., 2023: 8.0°; Wang et al., 2025: 8.4°).

The modest negative proportional bias (−0.22%) corroborates prior observations that external trunk morphology over‑represents mild curves and plateaus in severe deformities, suggesting that future calibration could further improve accuracy at the extremes. BMI analysis revealed no significant relationship between body habitus and prediction accuracy within the normal-to-overweight range (BMI 15.1–25.9, *r* = 0.15, *p* = 0.46). While the training cohort included the full adolescent BMI spectrum (14.3–41.8 kg/m^2^) including three obese patients, the test set lacked severely obese individuals (BMI ≥ 30), limiting validation in this subgroup despite their exposure during model training. Prospective validation in obese patients is warranted.

One limitation of this study is the relatively high number of rejected scans for poor quality. With more testing of the app, the research community may progressively better understand the circumstances leading to bad quality scans: unintended patient movement during the acquisition, operators’ speed of execution, operator–patient distance, etc. Scans also tend to lead to less accuracy in patients with higher BMI [10, 27]. Extra tissue may hide spinal deformities which in turn leads to inferior prediction. Finally, quality of prediction decreased in curves above 50° which may be the result of decreased association between internal and external rotational deformities. More severe spinal deformities may not correspond to proportionally greater trunk rotation [20, 28, 29]. Prediction is less accurate for larger curves due to the limited sample size, and especially because the dataset contains few cases of such curves; oversampling of larger curves may improve algorithm performance.

## Conclusion

The implementation of 3D topography combined with AI seems to improve the accuracy of the classic surface topography in predicting scoliotic Cobb angles. The app demonstrates its strongest performance in smaller curves, highlighting the necessity for further optimization in patients with larger deformities and higher BMI. This accessible smartphone-based tool has the potential to reshape scoliosis follow-up by enabling frequent at-home remote monitoring, reducing unnecessary hospital visits and radiographs, and facilitating early detection of curve progression.

## Supplementary Information

Below is the link to the electronic supplementary material.

Supplementary file1 (DOCX 2465 KB)

## Data Availability

Measurements and analyses done at the CHU Sainte-Justine are on a password-protected server. Access may be arranged through application to the REB.
