# Supplementary figures and images for: Artificial intelligence-driven 3D surface-topography app for screening and monitoring adolescent scoliosis: early results from a single institution
Source: Spine Deform. 2026 Jan 30;14(3):851–8. doi: 10.1007/s43390-026-01282-5 (PMC13282326; doi:10.1007/s43390-026-01282-5)

**Figures (Supplementary material):**


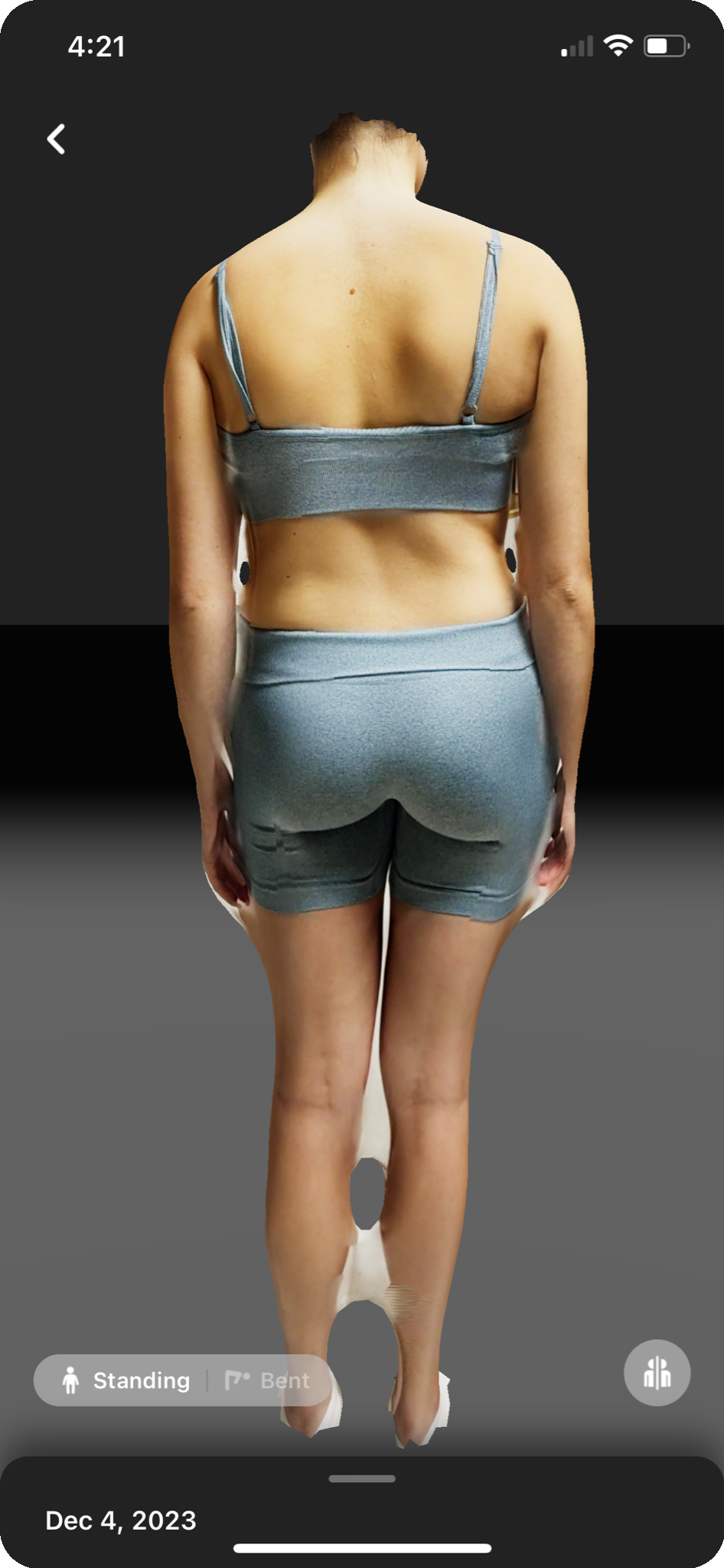


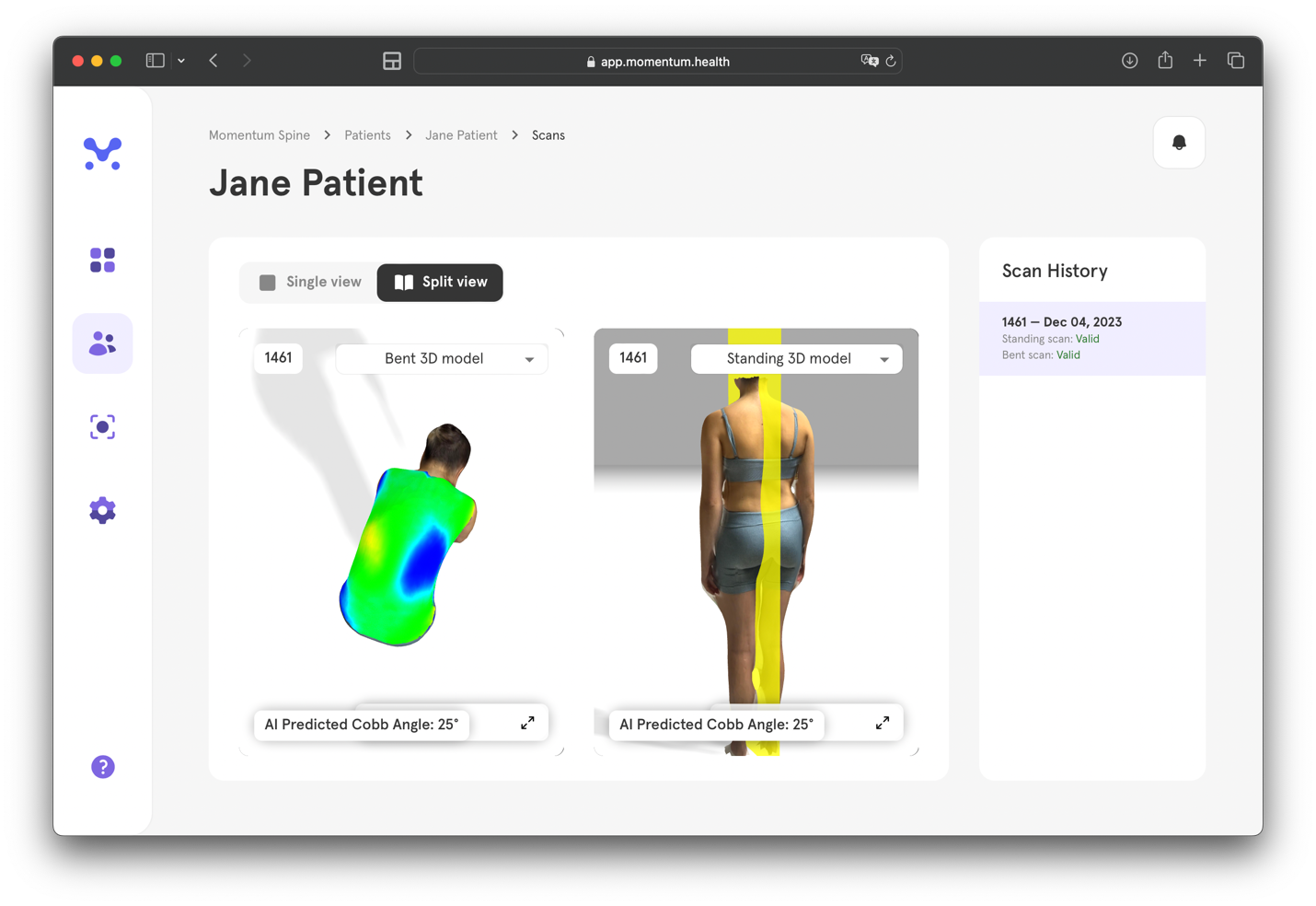

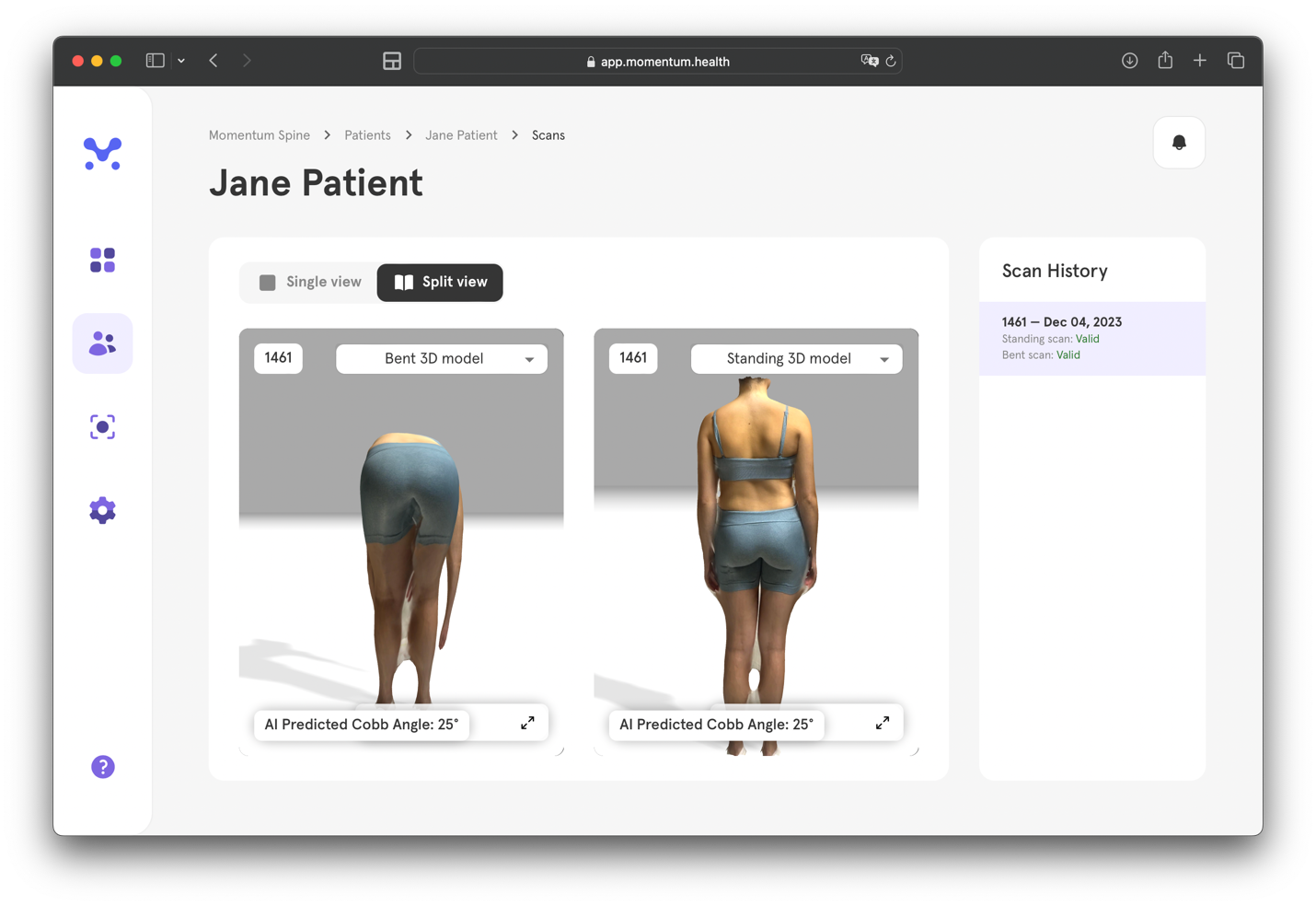

Supplement: Supplementary file 1 — Supplementary file1 (DOCX 2465 KB) [file 43390_2026_1282_MOESM1_ESM.docx]
